# The Impact of Comprehensive Case Management on HIV Client Outcomes

**DOI:** 10.1371/journal.pone.0148865

**Published:** 2016-02-05

**Authors:** Mark Brennan-Ing, Liz Seidel, Leslie Rodgers, Jerome Ernst, Doug Wirth, Daniel Tietz, Antonio Morretti, Stephen E. Karpiak

**Affiliations:** 1 Center on HIV and Aging, ACRIA, New York, New York, United States of America; 2 College of Nursing, New York University, New York, New York, United States of America; 3 Graduate School of Social Service, Fordham University, New York, New York, United States of America; 4 BOOM! Health, Bronx, New York, United States of America; 5 Amida Care, Inc., New York, New York, United States of America; 6 Human Resources Administration, City of New York, New York, New York, United States of America; 7 Columbia University, New York, New York, United States of America; Vanderbilt University, UNITED STATES

## Abstract

In 1990, New York State instituted Comprehensive Medicaid Case Management, also known as Target Case Management (TCM), for people dealing with multiple comorbid conditions, including HIV. The goal of TCM is to assist clients in navigating the health care system to increase care engagement and treatment adherence for individuals with complex needs. HIV-positive individuals engaged in care are more likely to be virally suppressed, improving clinical outcomes and decreasing chances of HIV transmission. The purpose of this study was to understand the impact of TCM management on outcomes for people with HIV. Data were obtained from Amida Care, which operates not-for-profit managed care Medicaid and Medicare Special Needs Plans (SNPs) for HIV clients. Changes in clinical, cost, as well as medical and pharmacy utilization data among TCM clients were examined between January 2011 through September 2012 from the start of case management enrollment through the end of the study period (i.e., up to 6 months after disenrollment). Additionally, CD4 counts were compared between Amida Care TCM clients and non-TCM clients. Notable findings include increased CD4 counts for TCM clients over the one-year study period, achieving parity with non-TCM clients (i.e., Mean CD4 count > 500). When looking exclusively at TCM clients, there were increases in medication costs over time, which were concomitant with increased care engagement. Current findings demonstrate that TCM is able to achieve its goals of improving care engagement and treatment adherence. Subsequent policy changes resulting from the Affordable Care Act and the New York State Medicaid Redesign have made the Health Home the administrator of TCM services. Government entities charged with securing and managing TCM and care coordination for people with HIV should provide thoughtful and reasonable guidance and oversight in order to maintain optimal clinical outcomes for TCM clients and reduce the transmission of HIV.

## Introduction

With Affordable Care Act (ACA) implementation and many of those with HIV accessing care through the expansion of Medicaid and access to other programs resulting from these reforms, there is concern about the fate of case management programs for those with HIV [1]. Due to ACA, there may be restrictions in case management and other supportive services for people with HIV which have been historically provided through the Ryan White Program [2]. For those concerned with HIV care delivery, losing support for “wrap around” non-medical services such as case management is contraindicated by the available evidence that these services are effective in keeping people with HIV engaged in care and adherent to treatments [3, 4].

Among the 1.2 million people in the U.S infected with HIV, only 40% are engaged in care, 37% prescribed anti-retroviral therapy (ARV), with 30% achieving the targeted clinical outcome of viral suppression [5]. Consequently, approximately 840,000 people in the U.S. are not receiving effective HIV care as evidenced by a lack of viral suppression. This finding is critical since those who are virally suppressed have better health outcomes and lower risk of HIV transmission [3]. Research shows that the epidemic could collapse through the reduction of individuals with high HIV viral loads who are the most likely to infect others [6–8]. The failure to achieve better rates of viral suppression can be attributed to a synergy of complex factors including behavioral health problems, unstable housing, incarceration, poor health literacy, and the economic, food, and housing insecurities endemic to poor communities of color which have the highest rates of HIV incidence [6–10].

### Case Management

Case management comprises a subset of care coordination models. Successful care coordination models demonstrate accountability for the organization of patient care, build respectful relationships and agreements among care partners, support patients regardless of where they access health care, and establish good communications among care partners [11–12]. During the early years of the HIV epidemic, New York State implemented case management services for people with HIV having complex care needs [13]. Case management uses a client-centered multi-step processes which, “ensures coordination and expedient access” to an array of medical and social supports [14], and acts as a megaservice that bridges HIV and non-HIV resources in complex and fragmented service environments [15]. Case management clients are more likely to have lower incomes and education, to be uninsured or publicly insured, to have a history of drug use, and to be racial/ethnic minorities, women or heterosexuals [14]. The goals of case management are to achieve care engagement and treatment adherence by helping the client to function independently through access to housing and other supportive services [16–18].

### Targeted Case Management in New York State

The Comprehensive Medicaid Case Management Program (also known as COBRA Case Management or Targeted Case Management [TCM]) began in 1990 in New York State. TCM targeted Medicaid-eligible populations, including HIV-infected persons, with multiple comorbid conditions including behavioral health issues. TCM utilizes a team of case managers and paraprofessionals to provide comprehensive intensive management services. TCM was designed for people with HIV who require frequent contact with care providers and have difficulty accessing and sustaining medical and supportive services. The goals of TCM are to: 1) provide access to services that foster independence and self-sufficiency; 2) ensure adherence to care and treatment; 3) prevent or delay institutionalization; 4) increase universal access to HIV-related services; and 5) promote early intervention—disease prevention [19]. The TCM program has served approximately 14,000 people with HIV [20]. To-date case management programs, including TCM, have demonstrated positive outcomes in terms of increased attention to client needs and an uptake in related medical and social services, better care engagement, improved ARV prescription rates and adherence, and a significant increase in CD4 counts between first and second assessment s (median = 6.2 months, range = 2.3 to 26.8 months) [17, 21].

### Purpose and Rationale

We sought to examine the effectiveness of TCM services for people with HIV in New York State with regard to increased care engagement and improved treatment adherence by analyzing changes in clinical, cost, and utilization data among TCM clients over the course of their case management enrollment.

## Materials and Methods

### Source of Data and Procedures

Data were obtained from Amida Care. Amida Care operates not-for-profit Medicaid and Medicare HIV Special Needs Plans (SNPs), providing managed care to HIV-positive people in New York City. Amida Care’s mission is to provide comprehensive care and coordinated services that facilitate positive health outcomes for its over 6,000 members. Amida Care was founded by seven AIDS Service Organizations in New York City (Community Health Network, Harlem United, Housing Works, Project Samaritan AIDS Services, Inc., Promesa, Inc., St. Mary's Episcopal Center, Inc., and Village Care).

The Amida Care database provides a consolidated source of member and claims information which is permanently maintained (i.e., HIV status, case management needs, contacts with the plan, outreach worker contacts, care coordination clinical notes, and CD4 count). Data on all members receiving TCM as determined by billing and procedure codes were extracted from the Amida Care database to provide the information for the analyses described below (N = 2072), from the start of the study period (January 2011) to the end of the study period (September, 2012). This study period was chosen to examine case management outcomes in the period immediately preceding the implementation of Medicaid Redesign in New York State in September, 2012. This time period was selected as it occurred prior to the major reorganization of TCM delivery systems through Medicaid Redesign, as such major systematic change had the potential to affect the provision and efficacy of TCM, as well as our ability to examine the effectiveness of TCM on client outcomes during Medicaid reorganization.

Prior to analyses, all data were de-identified. Research protocols were approved by the Gay Men’s Health Crisis (GMHC) Institutional Review Board. Because this was a secondary analysis of de-identified data, no consent was obtained. The average age of the TCM sample was 48.0 years, 33% were women and 67% were men.

### Measures

To better understand the impact of TCM on clients over time, cost, utilization and clinical outcomes were examined. These included actuarial risk scores derived from the IMPACT PRO proprietary software package, which provides an estimation of future member medical costs based on patient age, gender, physical and mental health comorbidities, and levels of service utilization [22]. This system employs multidimensional episode-based predictive modeling through the combination of clinical and administrative claims data. Short-term change in risk scores can be driven by greater service utilization and newly diagnosed comorbid conditions. We also examined actual annualized patient total care and medication costs. To assess care engagement and treatment adherence, we examined utilization (number of emergency room, inpatient hospital, outpatient hospital, mental health, and primary care visits) and prescription fills for ARVs and psychotropic medication. We focused on ARVs and psychotropic medications since improving HIV treatment adherence and behavioral health care are the main targets of the TCM program, and these were the medication types most likely to show change due to TCM. With regard to utilization measures, we also calculated the ratio of each type of utilization to total health care utilization (e.g., Emergency Room Ratio = Number Emergency Room Visits/Total Number of Health Care Visits) to assess the intensity of each type of utilization, as well as the median time between visits to assess frequency of utilization. Lastly, we examined CD4 T-cell counts to gauge the effects of improved care engagement and treatment adherence resulting from TCM engagement.

### Design and Analysis

The present paper consisted of an observational study of Amida Care members receiving TCM during the study period. TCM clients are not homogenous and may utilize case management services differently with regard to volume, frequency and duration. To account for this heterogeneity and to identify patterns in case management utilization, cluster analysis was performed with the goal of identifying groupings such that TCM clients within the same group utilized TCM services similarly, and clients between groups displayed significantly different case management utilization patterns. Three indicators of case management utilization were used to cluster members: 1) the total number of TCM visits during the study period; 2) the median time between consecutive TCM visits; and 3) the time between the first and final case management visits. These indicators were chosen to capture the temporal nature of the data of volume, frequency and duration of TCM services, respectively. The k-means algorithm with a four-cluster solution provided the best interpretability of utilization patterns. This analysis did not address utilization of non-TCM services.

Statistically significant change over time was examined with repeated-measures MANOVA, with the TCM utilization pattern typology derived from cluster analysis utilized as a between-subjects factor to examine if categorical differences in TCM utilization were related to study outcomes. The examination of IMPACT PRO actuarial risk scores, total costs, and medication costs, examined these variables from the start of TCM enrollment to the end of the study period. Analysis of change in prescription medication fills (ARVs and psychotropics) considered the number of prescriptions filled during the first 3 months of the study period (January, February and March 2011) compared with the final 3 months (July, August, and September, 2012). The service utilization analysis examined the total number of services by provider type and TCM utilization pattern for the entire study period. The analysis of change in CD4 counts examined 3 time points: 1) TCM Enrollment; 2) TCM case closure; and 3) End of Study Period (September 30, 2012). The study period comprised 638 days. Overall, the average median duration of TCM enrollment from the start of the study period to TCM case closure was 224 days.

We also compared TCM members to all Amida Care members not receiving TCM (N = 937) who had CD4 values available during the study period (i.e., beginning, mid-point, and end) using a quasi-experimental design of pre-existing groups. Both TCM members and non-TCM members were HIV-positive and eligible for Medicaid. This analysis examined the impact of TCM participation on this indicator of immune function using repeated-measures MANOVA (TCM yes/no X Time) to assess the relative impact of case management services in the context of this population.

## Results

### TCM Utilization Patterns

Four independent patterns of TCM utilization emerged from the cluster analysis. The four orthogonal groupings were labeled as long-term moderate-intensity, moderate-term moderate-intensity, short-term low-intensity and short-term high-intensity. Long-term moderate-intensity TCM clients (i.e. Long-Moderate) evidenced higher means for duration of TCM service utilization during the study period and average median times between consecutive TCM visits (i.e., intensity). The moderate-term moderate-intensity group (i.e., Moderate-Moderate) exhibited an average number of TCM visits, duration of time in care, and average median times between consecutive visits. Clients within short-term low-intensity (i.e., Low Intensity) did not use TCM services for a long period of time and had a low number of service visits. The short-term high-intensity group (i.e., High Intensity) displayed a high number of TCM visits during a short duration of time.

### Change in Outcomes over Time

#### *Impact Pro* Actuarial Risk Scores

Among TCM clients, there were significant differences in participants estimated healthcare utilization costs as indicated by *Impact Pro* actuarial risk scores by type of TCM utilization, and a significant increase in estimated costs from first to final average risk scores (Table 1). This increase over time was observed in all four groups; Long-Moderate (7.1 vs. 8.6), Moderate-Moderate: (7.5 vs. 8.6), Low Intensity (8.3 vs. 9.5), and High intensity (7.3 vs. 8.5). Collapsing across all TCM utilization groups, the average actuarial score rose from 7.4 to 8.7 at the end of the study period. This short-term change in estimated healthcare utilization costs is consistent with greater service use and care engagement during the period of TCM enrollment, which is in line with the goal of the TCM program.

**Table 1:** Change in IMPACT PRO Risk Scores over TCM Enrollment Period.

		First Risk Score		Final Risk Score	
	N	Mean	SD	Mean	SD
Long-Moderate	471	7.06	4.73	8.62	4.86
Moderate-Moderate	169	7.49	4.12	8.55	4.03
Low Intensity	178	8.33	4.18	9.47	4.31
High Intensity	441	7.26	3.59	8.49	4.40
Total	1259	7.36	4.21	8.68	4.52

*Note*. Multivariate: F (7,2708) = 11.82, *p* < .001. Differences by Time: F (1,2708) = 43.55, *p* < .001. Differences by TCM Pattern Group: F (3,2708) = 6.05, *p* < .001. TCM Group X Time: F (3,2708) = 0.46, NS.

#### Medication and Total Costs

Medication costs among TCM participants differed significantly by utilization pattern and increased significantly from initial to final costs ($10, 857 and $28,590, respectively). Total Costs were significantly increased over time all TCM utilization groups, on average $20,537 rising to $38,404 (Table 2). The increase in average total costs was observed in each of the four groups; Long-Moderate ($20,171 vs. $37,775), Moderate-Moderate: ($20,582 vs. $38,028), Low Intensity ($22,633 vs. $42,076), and High Intensity ($20,162 vs. $37,796). The increased total costs over the study period can be attributed to increased health care utilization and medication use.

**Table 2:** Change in Total Costs and Medication Costs over TCM Enrollment Period.

		Initial Costs ($)				Final Costs ($)			
		Medication		Total		Medication		Total	
	N	Mean	SD	Mean	SD	Mean	SD	Mean	SD
Long-Moderate	471	11,441	13,761	20,171	22,566	28,066	15,378	37,755	27,493
Moderate-Moderate	169	9,792	9,854	20,582	24,979	28,187	17,490	38,028	25,472
Low Intensity	178	9,616	7,369	22,633	22,297	28,617	13,147	42,076	32,800
High Intensity	441	11,071	11,510	20,162	19,374	29,287	29,682	37,796	39,825
Total	1,259	10,857	11,791	20,537	21,726	28,590	21,461	38,404	32,788

*Note*. Multivariate: F (14,5414) = 54.64, *p* < .001. Differences by Time: F (2,2707) = 320.13, *p* < .001. Differences by TCM Pattern Group: F (6,5414) = 4.10, *p* < .001. TCM Group X Time: F (6,5414) = 0.68, NS.

#### Health Care Visit Frequency

Among TCM clients there were significant differences in the number of medical visits based on TCM utilization type (Table 3). Low Intensity had the highest average trips to the emergency room (2.5) compared to all other groups: Long-Moderate (1.5), Moderate-Moderate (1.5), and High Intensity (1.7). Low Intensity also had the most in-patient visits compared to the other groups (1.1). Long-Moderate and Moderate-Moderate users had the highest number of mental health visits (7.2 and 7.0, respectively) as compared with Low Intensity (6.2) and High Intensity groups (5.7). With regards to outpatient hospital visits, averages were similar for Long-Moderate (2.0), Moderate-Moderate (1.9), High Intensity (2.0), and were lowest for Low Intensity clients (0.8). Low Intensity had the most primary care visits (14.2) followed by: Moderate-Moderate (12.9), High Intensity (12.7), and Long-Moderate (12.0). There were no significant differences in ratios of visits by visit type among TCM utilization groups, suggesting that emergency room, in-patient, mental health, out-patient, and primary care visits were used with equal intensity by all TCM clients (Table 4). Additionally, there were no significant differences in median time between medical visits for any type of services (Table 5).

**Table 3:** Number of Visits by Type: Emergency Room, Inpatient, Mental Health, Out-patient and Primary Care.

Type of Visit	Long-Moderate		Moderate-Moderate		Low Intensity		High Intensity		Total	
	M	SD	M	SD	M	SD	M	SD	M	SD
Emergency Room	1.48	2.61	1.46	2.59	2.53	3.64	1.74	2.93	1.70	2.88
In-patient	.85	2.09	.91	1.80	1.06	2.00	.94	2.13	.91	2.06
Mental Health	7.16	13.49	6.96	11.08	6.16	7.68	5.71	11.37	6.52	11.92
Out-patient	1.96	5.30	1.91	5.06	.76	1.87	2.01	5.06	1.82	4.89
Primary Care	12.01	8.60	12.85	8.72	14.19	8.08	12.73	8.16	12.62	8.41

*Note*. Long-Moderate N = 360; Moderate-Moderate N = 92; Low Intensity N = 108; High Intensity N = 283; Total N = 843. Multivariate: F (15,2511) = 2.13, *p* < .01.

**Table 4:** Ratio of Visits by Type of Visit: Emergency Room, In-patient, Mental Health, Out-patient, and Primary Care.

Type of Visit	Long-Moderate		Moderate-Moderate		Low Intensity		High Intensity		Total	
	M	SD	M	SD	M	SD	M	SD	M	SD
Emergency Room	.08	.15	.07	.11	.11	.16	.08	.13	.08	.14
In-patient	.04	.09	.03	.07	.04	.08	.04	.07	.04	.08
Mental Health	.19	.21	.20	.22	.19	.18	.17	.20	.19	.1
Out-patient	.05	.10	.04	.10	.02	.05	.06	.13	.05	.12
Primary Care	.65	.28	.65	.28	.63	.24	.65	.27	.65	.27

*Note*. Long-Moderate N = 360; Moderate-Moderate N = 92; Low Intensity N = 108; High Intensity N = 283; Total N = 843. Multivariate: F (12,2514) = 1.73, NS.

**Table 5:** Median Time between Visits by Type of Visit: Emergency Room, In-patient, Mental Health, Out-patient, and Primary Care.

Type of Visit	Long-Moderate			Moderate-Moderate			Low Intensity			High Intensity			Total		
	N	M	SD	N	M	SD	N	M	SD	N	M	SD	N	M	SD
All Kinds of Visits	355	20.8	17.1	91	22.7	16.8	108	20.2	13.9	283	21.1	16.7	837	21.0	16.6
Emerg-ency Room	231	30.3	23.6	58	35.1	24.1	83	30.9	23.5	166	32.4	24.9	538	31.6	24.0
In-patient	219	29.4	25.3	52	31.5	26.2	67	37.6	27.9	159	29.0	25.0	497	30.6	25.8
Mental Health	300	26.8	22.3	66	24.4	19.0	95	25.7	22.6	226	25.5	20.5	687	26.0	21.4
Out-patient	285	16.9	16.5	71	16.7	16.0	72	18.8	15.9	207	19.4	18.3	608	18.0	17.0
Primary Care	351	31.6	18.2	88	32.9	16.2	104	32.0	14.8	275	35.0	20.3	818	33.0	18.5

*Note*. All Kinds of Visits: F (3,833) = .43, NS. Emergency Room: F (3,534) = .71, NS. In-patient: F (3,493) = 2.05, NS. Mental Health: F (3,683) = .31, NS. Out-patient: F (3,604) = .98, NS. Primary Care: F (3,814) = 1.87, NS.

#### Prescription of ARV and Psychotropic Medications

The number of psychotropic prescriptions filled during the first 3 months and the last 3 months did not change significantly (0.5 and 0.5, respectively), and the average number did not differ significantly by intensity group (Table 6). There was not a significant difference between changes in prescriptions for ARV medications from the first 3 months to the last 3 months (4.6 and 4.9, respectively), but there were differences by TCM utilization group; Low Intensity clients reported the greatest number of ARV prescriptions on average during both periods (Table 7). Thus, we did not observe any significant change in ARV or psychotropic medication use over the course of TCM enrollment, but the number of ARV prescriptions was related to the type of TCM utilization. Because the number of ARV and psychotropic prescriptions did not change during TCM enrollment, the change in medication costs reported earlier are likely due to an increase in non-ARV/psychotropic medications prescribed for other comorbid conditions.

**Table 6:** Change in Prescriptions for Psychotropic: First 3 Months vs. Last 3 Months.

		First 3 Months		Last 3 Months	
	N	Mean	SD	Mean	SD
Long-Moderate	727	0.39	1.25	0.45	1.33
Moderate-Moderate	185	0.58	1.70	0.52	1.61
Low Intensity	207	0.58	1.59	0.36	1.01
High Intensity	605	0.56	1.48	0.53	1.36
Total	1926	0.45	1.36	0.46	1.30

*Note*. Multivariate: F (9,3446) = 1.33, *p* = .22. Differences by Time: F (1,3446) = 0.08, *p* = .78. Differences by TCM Pattern Group: F (4,3446) = 2.18, *p* = .07. TCM Group X Time: F (4,3446) = 0.71, *p* = .59.

**Table 7:** Change in Prescriptions for ARV Medications: First 3 Months vs. Last 3 Months.

		First 3 Months		Last 3 Months	
	N	Mean	SD	Mean	SD
Long-Moderate	727	4.39	4.40	4.80	3.98
Moderate-Moderate	185	5.75	4.07	5.16	3.94
Low Intensity	207	6.18	4.64	5.49	3.88
High Intensity	605	4.96	4.20	4.98	4.07
Total	1926	4.55	4.41	4.89	4.01

*Note*. Multivariate: F (9,3446) = 54.64, *p* < .001. Differences by Time: F (1,3446) = 0.98, *p* = .32. Differences by TCM Pattern Group: F (4,3446) = 8.36, *p* < .001. TCM Group X Time: F (4,3446) = 1.87, *p* = .11.

#### Relationship between CD4 T-Cell Counts and Time of TCM Enrollment

There was a significant increase in CD4 Count from enrollment in TCM, to TCM case closure, and to the end of the study period (288.7, 295.8, and 503.0, respectively). On average, the first CD4 measurement occurred in 244 days after the start of the study period. The last CD4 measurement while in TCM was at 523 days on average, or a difference on average of 279 days between first and last CD4 count measurement while enrolled in TCM. The average difference between the last TCM CD4 measurement and the end of the study period was 115 days. This increase in CD4 count from the beginning to end of the study period was observed in TCM utilization groups; Long-Moderate (295 vs. 500), Moderate-Moderate (278 vs. 545), Low Intensity (299 vs. 462) and High Intensity (289 vs. 503). Changes in CD4 count over time did not vary significantly by TCM utilization patterns (Table 8).

**Table 8:** Change in CD4 Count over TCM Enrollment and Follow-up Period: TCM Clients.

		First CD4		Intermediate CD4		Final Study CD4	
	N	Mean	SD	Mean	SD	Mean	SD
Long-Moderate	356	295.06	159.89	299.22	165.93	500.29	323.61
Moderate-Moderate	91	277.70	162.18	282.13	163.09	545.37	306.63
Low Intensity	108	298.89	159.84	313.07	156.81	462.71	281.41
High Intensity	283	280.45	161.75	289.39	155.33	508.01	321.77
Total	838	288.74	160.68	295.83	160.90	502.95	316.15

*Note*. Multivariate: F (11,2839) = 39.31, *p* < .001. Differences by Time: F (2,2839) = 157.31, *p* < .001. Differences by TCM Pattern Group: F (3,2839) = 0.38, NS. TCM Group X Time: F (6,2839) = 1.34, NS.

#### Comparison of CD4 Counts: TCM and Non-TCM Clients

When comparing changes in average CD4 counts, we included age and gender as covariates in preliminary analysis as these factors differed significantly between those enrolled in TCM versus those not enrolled in TCM. TCM members compared to non-TCM members were significantly older on average, 48 and 47 years, respectively [*t*(3027) = 3.37, *p* < .001], and had a lower proportion of men, 67% and 77%, respectively [*X*^*2*^(1) = 29.67, *p* < .001]. However, neither age nor gender exhibited significant effects in the multivariate model (*p* = .19 and *p* = .79, respectively), so we removed these factors from the analysis and report the unadjusted means below. Non-TCM clients had significantly higher CD4 levels compared to TCM clients at first and intermediate assessments (514.4 vs 288.7, and 515.2 vs 295.8, respectively). However, by the end of the study period the TCM group on average had achieved near parity with the non-TCM group with CD4 counts of 503.0 and 525.1, respectively (Table 9).

**Table 9:** Comparison of Change in CD4 Count over TCM Enrollment: TCM and Non-TCM Clients.

		First CD4		Intermediate CD4		Final Study CD4	
	N	Mean	SD	Mean	SD	Mean	SD
TCM Clients	838	288.74	160.68	295.83	160.90	502.95	316.15
Non-TCM Clients	937	514.40	287.59	515.20	286.77	525.14	287.15

*Note*. Multivariate F (5,5656) = 161.62, *p* < .001. Differences by Time: F (2,5656) = 91.99, *p* < .001. Differences by TCM Utilization Group: F (1,5656) = 539.78, *p* < .001. TCM Group X Time: F (2,5656) = 73.76, *p* < .001.

## Discussion

Our examination of case management utilization patterns revealed four distinct typologies; typologies that differed based on the frequency, volume and duration of TCM engagement. These patterns suggest, in part, that TCM services had been responsive to variable client needs, and it is likely that this typology is related to client characteristics, including comorbid health conditions, behavioral health issues, and housing instability. For example, Low Intensity clients had the highest average actuarial risk scores and total costs both before and after TCM engagement. This group also had the highest levels of emergency room, inpatient hospital, and primary care utilization, but were among the lowest users of mental health services. Taken together these findings suggest that the Low Intensity TCM clients may represent the most complex cases among this cohort of TCM users, which may be related to their relative lack of engagement in case management services and behavioral health care compared to the other three groups.

With regard to changes over time as a whole, findings support the hypothesis that TCM improves clients’ engagement with care and treatment adherence as evidenced by significant increases in actuarial risk scores (i.e., a proxy for increased service utilization), medication costs, and total costs. Given that we did not observe a significant change in the number of psychotropic or ARV medications, increased medication costs can be attributed to treatment for other comorbid conditions. This is a positive finding as it implies better screening and diagnosis of comorbidities through engagement with health care providers. The most telling evidence, however, was the significant increase in average CD4 T-cell counts over the study period with the TCM group reaching parity with non-TCM members. Since we did not observe that TCM clients filled more ARV prescriptions following enrollment, the most likely explanation is that they were more adherent and/or may have been placed on more efficacious ARVs through regular engagement with health care providers as indicated by the restoration of immune function.

## Limitations

While the current study has provided important evidence regarding the effectiveness of TCM, our study is limited as we examined clients from only one managed care plan, and findings may not be generalizable to all clients receiving TCM. In addition, demographic characteristics of the sample were restricted to age and gender, which did not allow analysis of how race/ethnicity and other factors may have been related to case management client outcomes. We also did not have data available on viral load or the specific ARV regimens that were prescribed to clients, which could have provided additional evidence regarding the efficacy of TCM.

### Policy and Program Implications

As the population with HIV grows older, their need for supportive services are likely to grow [22–25], rendering the types of support provided by TCM increasingly important.

#### Medicaid Redesign and Targeted Case Management in New York State

The New York State Medicaid Redesign effort was launched in 2011 to ensure future fiscal sustainability of this program, and is having a substantial impact on the way services are delivered and reimbursed for the almost 130,000 New Yorkers living with HIV. These reforms include developing Health Homes supported through ACA funds [26], and adopting the Health Home model of service delivery to provide case management. The Health Home model represents a paradigm shift from a focus on episodic illness care to a coordinated care model that encompasses acute, chronic and preventive care across the lifespan [2, 12, 16].

However, relocating case management services into Health Homes that may have little experience meeting the complex needs of people with HIV may be problematic. Failure to keep these clients adherent to ARVs and engaged in care would likely result in a cascade of poor health outcomes, increased medical expenditures due to increased morbidity, and other costs. Better control of HIV disease through enhanced case engagement should improve clinical outcomes which would result in long-term cost savings [3]. Aside from individual quality-of-life concerns, these sequelae would severely undercut the goal of reducing Medicaid costs. Initial data show that for New York State Medicaid patients *without* prior case management, Health Home enrollment is associated with decreased emergency room visits and hospital admissions, and increased primary care visits [27]. However, no data are available to assess the impact Health Home enrollment on clients who had been receiving TCM, so it is difficult to evaluate the success of this model at present.

#### National HIV/AIDS Strategy and “Ending AIDS”

As noted in the National HIV/AIDS Strategy (NHAS) successful HIV treatment results in lower community viral loads, leading to a lower incidence and prevalence of HIV [28]. HIV Case management is an important tool in implementing the NHAS, and is also crucial to current efforts to “End AIDS” which involve expanded testing, care engagement, and ARV adherence to control and prevent HIV infection [29]. Reasonable regulatory expectations and oversight ought to recognize that what were formerly TCM clients with HIV remain a special population whose complex needs cannot be met by a “one size fits all” approach [30].

As greater numbers of people with HIV secure health insurance coverage through the ACA’s health exchanges and Medicaid expansion, increased attention to successful care engagement, focused treatment adherence, and effective care coordination models are required from stakeholders, including states, insurance plans, medical and social service providers, and beneficiaries. State Medicaid directors and health plan executives will see benefits from valuing targeted services that facilitate access and retention in care by those individuals with the most complex physical and behavioral health care.

Since many states do not invest Medicaid dollars in the full range of services necessary to achieve better health outcomes, flexible federal funding streams such as Ryan White will be critical to not only achieving the NHAS, but to redesigning health care systems to insure better health outcomes and mitigate expenditures [3]. By advancing effective engagement and retention in care, such as New York State’s TCM program, stakeholders reap interrelated and compounded benefits including better health outcomes, avoiding costly long-term care services, increasing the value of ongoing medication and treatment expenditures, and preventing HIV infection by reducing community levels of viral load. By preventing new infections, New York State alone stands to avoid future per patient lifetime HIV care costs totaling over $400,000. In order to achieve these goals, investing in care coordination services for individuals with most complex needs such as HIV, should not be reduced in an effort to address the needs of other emerging populations.

## Conclusions

A recent study of health care providers underscores concerns as case management for people with HIV migrates to the Health Home model [31], including continuity of care, sensitivity to the client with HIV, and providers’ understanding of the constellation of stressors such as mental illness, substance use, poverty, and endemic stigma that affect this population. Sensible and stakeholder-driven Medicaid redesign and the implementation of Health Home model represents an important opportunity to remake a significant part of the health and human services delivery system serving people with HIV. This effort could serve as a model to other states seeking to reduce their Medicaid and other health care expenditures. In this process, however, we urge careful attention to vulnerable, high-needs individuals, such as people with HIV who require TCM. In 2012 the International Association of Physicians in AIDS Care, examined data from 325 published studies, and provided guidelines for improving entry, retention in care, and adherence [32]. One of the primary recommendations was the need for “strengths-based case management.” In our view, such careful attention includes providing thoughtful and reasonable guidance and oversight by government entities charged with securing and managing comprehensive case management and care coordination for people with HIV.
